# Left-right olfactory asymmetry results from antagonistic functions of voltage-activated calcium channels and the Raw repeat protein OLRN-1 in *C. elegans*

**DOI:** 10.1186/1749-8104-2-24

**Published:** 2007-11-06

**Authors:** Sarah L Bauer Huang, Yasunori Saheki, Miri K VanHoven, Ichiro Torayama, Takeshi Ishihara, Isao Katsura, Alexander van der Linden, Piali Sengupta, Cornelia I Bargmann

**Affiliations:** 1 Howard Hughes Medical Institute and Rockefeller University, New York, NY 10065, USA; 2 Herbert W Boyer Program in Biological Sciences, The University of California, San Francisco, San Francisco, CA 94143, USA; 3 Structural Biology Center, National Institute of Genetics, and Department of Genetics, The Graduate University for Advanced Studies, Mishima, 411-8540, Japan; 4 Department of Biology and National Center for Behavioral Genomics, Brandeis University, Waltham, MA 02454, USA

## Abstract

**Background:**

The left and right AWC olfactory neurons in *Caenorhabditis elegans *differ in their functions and in their expression of chemosensory receptor genes; in each animal, one AWC randomly takes on one identity, designated AWC^OFF^, and the contralateral AWC becomes AWC^ON^. Signaling between AWC neurons induces left-right asymmetry through a gap junction network and a claudin-related protein, which inhibit a calcium-regulated MAP kinase pathway in the neuron that becomes AWC^ON^.

**Results:**

We show here that the asymmetry gene *olrn-1 *acts downstream of the gap junction and claudin genes to inhibit the calcium-MAP kinase pathway in AWC^ON^. OLRN-1, a protein with potential membrane-association domains, is related to the *Drosophila *Raw protein, a negative regulator of JNK mitogen-activated protein (MAP) kinase signaling. *olrn-1 *opposes the action of two voltage-activated calcium channel homologs, *unc-2 *(CaV2) and *egl-19 *(CaV1), which act together to stimulate the calcium/calmodulin-dependent kinase CaMKII and the MAP kinase pathway. Calcium channel activity is essential in AWC^OFF^, and the two AWC neurons coordinate left-right asymmetry using signals from the calcium channels and signals from *olrn-1*.

**Conclusion:**

*olrn-1 *and voltage-activated calcium channels are mediators and targets of AWC signaling that act at the transition between a multicellular signaling network and cell-autonomous execution of the decision. We suggest that the asymmetry decision in AWC results from the intercellular coupling of voltage-regulated channels, whose cross-regulation generates distinct calcium signals in the left and right AWC neurons. The interpretation of these signals by the kinase cascade initiates the sustained difference between the two cells.

## Background

Olfactory neurons sense environmental chemicals using large families of chemoreceptor genes that are deployed in elaborate patterns. For example, the main olfactory organs of the nematode *Caenorhabditis elegans *are the bilateral (left and right) amphids, which house 12 pairs of ciliated sensory neurons. Each sensory neuron expresses many receptor genes, in contrast with vertebrate olfactory neurons that generally express one receptor gene per cell [1]. Every neuron pair expresses a unique complement of receptors, and in addition, expression of some receptor genes is asymmetric on the left and right sides. For example, the left and right ASE gustatory neurons (ASEL and ASER) are structurally similar, but they express different receptor genes and sense different tastants [2,3]. ASE asymmetry, which is established by transcription factors and microRNAs, is stereotyped and tightly coupled to the body plan [4]. The left and right AWC olfactory neurons also have distinct functions, but AWC asymmetry is variable [5]. The receptor gene *str-2 *is expressed stochastically in one of the AWC neurons, such that half of the animals express *str-2 *in AWCL and half of the animals express *str-2 *in AWCR. The AWC cell that expresses *str-2 *is designated AWC^ON^, and the cell lacking *str-2 *expression is designated AWC^OFF^. These alternative AWC gene expression patterns correlate with different olfactory functions: both AWCs sense the odors benzaldehyde and isoamyl alcohol, but only AWC^ON ^senses butanone, and only AWC^OFF ^senses 2,3-pentanedione [6]. Behavioral analysis of animals with an altered complement of AWCs has demonstrated that AWC asymmetry increases olfactory discrimination and olfactory plasticity [6,7].

Signaling between the two AWCs is required for the diversification of AWC^ON ^and AWC^OFF^. If one AWC precursor is killed in the embryo, the surviving cell always becomes AWC^OFF^, suggesting that AWC^OFF^ is a ground state and AWC^ON ^is an induced state [5]. The random left-right specification and signaling between equipotential AWCs are reminiscent of developmental lateral signaling, which is usually mediated by Notch receptors and Delta/Serrate ligands [8], but Notch pathway genes have no apparent role in AWC asymmetry [5]. Instead, the induction of AWC^ON ^requires NSY-5, an innexin gap junction protein, and NSY-4, a protein similar to claudins and the regulatory γ subunits of voltage-activated calcium channels [9,10]. NSY-5 creates a transient gap junction network essential for communication between the left and right AWCs. NSY-5-dependent ultrastructural gap junctions link the cell bodies of the embryonic AWC neurons with many additional neurons; these gap junctions disappear soon after hatching [9]. Genetic experiments indicate that AWC^ON ^induction involves contributions from both the left and right AWC neurons as well as other neurons in the network. *nsy-4*, which is related to proteins that regulate channels and cell adhesion, also has network functions – it has cell-autonomous effects within the AWC neuron that expresses it, and cell non-autonomous effects on the contralateral AWC [10]. Even in a wild-type genetic background, the level of *nsy-4 *or *nsy-5 *activity in one AWC neuron is sensed by the contralateral AWC, so that the neuron with higher *nsy-4 *or *nsy-5 *expression preferentially becomes AWC^ON^. It is likely that the networks on the left and right are linked in the nerve ring, where axons from the left and right sides meet [5].

*nsy-5 *and *nsy-4 *induce AWC^ON ^by repressing a kinase cascade that includes the calcium/calmodulin-dependent kinase II (CaMKII) UNC-43, the p38/JNK mitogen-activated protein kinase kinase kinase (MAPKKK) NSY-1/ASK-1, and the MAPKK SEK-1, along with the signaling scaffold protein TIR-1 [5,11-13]. The downstream kinase cascade behaves straightforwardly and cell-autonomously: a cell with high activity of the kinase homologs becomes AWC^OFF ^and a cell with low gene activity becomes AWC^ON^, regardless of the kinase activity in the contralateral AWC [11,12].

The results described above define a decision point between the *nsy-5/nsy-4 *multicellular signaling network and cell-autonomous execution of the decision by the kinase homologs. In previous studies, two genes encoding subunits of a CaV2-type voltage-activated calcium channel were shown to affect AWC asymmetry upstream of the kinases [5]. Loss-of-function mutants in *unc-2*, the CaV2 pore-forming α1 subunit, result in a mixed phenotype with wild-type, 2AWC^ON^, and 2AWC^OFF ^animals. By contrast, mutants in the regulatory α2δ subunit *unc-36 *have a pure, strong 2AWC^ON ^phenotype; the reason for this difference was unknown. Here we show that *unc-2 *cooperates with a second calcium channel α1 subunit, the CaV1 homolog *egl-19*, explaining the difference between *unc-2 *and *unc-36*. Rescue experiments and mosaic analysis provide evidence that the calcium channels act in cell communication between AWCs. The activity of the calcium channels is opposed by the AWC asymmetry gene *olrn-1*, which acts downstream of *nsy-5 *and *nsy-4 *in the induction of AWC^ON^. *olrn-1 *represses the CaMKII/MAPK kinase cascade in AWC^ON ^and provides feedback to AWC^OFF^, coordinating the decisions of the left and right AWCs.

## Results

### The *olrn-1 *gene is expressed in AWC and promotes induction of AWC^ON^

**Figure 1:**
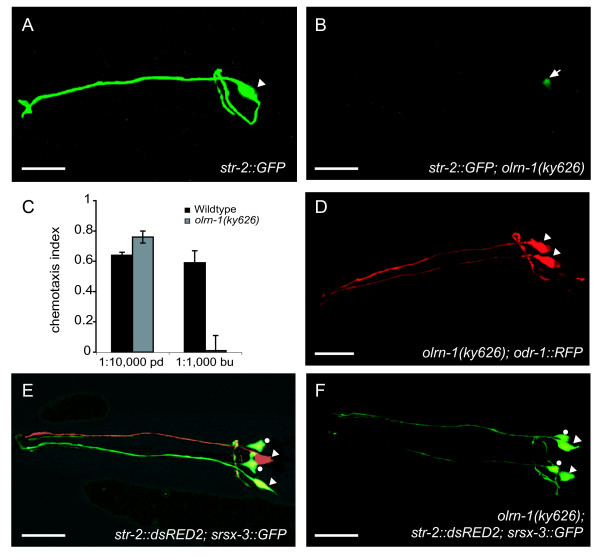
*olrn-1 *mutants have two AWC^OFF ^neurons. **(a,b) ***str-2::GFP *expression in (a) wild type and (b) *olrn-1(ky626) *animals. Arrowhead, AWC; arrow, dim *str-2::GFP *expression in ASI. **(c) **Chemotaxis of wild-type and *olrn-1(ky626) *animals to the AWC^OFF ^sensed odorant 2,3-pentanedione (pd) and the AWC^ON ^sensed odorant butanone (bu). A chemotaxis index of 1 indicates that 100% of animals approached the odorant, while a chemotaxis index of 0 represents random behavior. Error bars indicate standard error of the mean. **(d) ***odr-1::DsRed *is expressed in both AWC neurons (arrowheads) of *olrn-1 *mutants, a pattern identical to the wild-type pattern. **(e,f) ***str-2::DsRed; srsx-3::GFP *expression in (e) wild type and (f) *olrn-1(ky626) *mutant animals. Arrowheads indicate AWCs; dots indicate AWBs. Scale bars, 20 μm. Images are stacked confocal images.

In a screen for AWC asymmetry mutants, we isolated the mutation *olrn-1(ky626)*, which was named based on the isolation of another allele, *olrn-1(ut305)*, from an olfactory learning screen [7]. *olrn-1(ky626) *animals had a highly penetrant 2AWC^OFF ^phenotype (Figure 1a,b, Table 1), and they were able to chemotax to 2,3-pentanedione, an odor sensed by AWC^OFF^, but not butanone, an odor sensed by AWC^ON ^(Figure 1c). The AWC cell fate marker *odr-1::dsRed *was normally expressed in both AWCs of *olrn-1 *mutants, and AWC axon guidance was also apparently normal (Figure 1d). These results suggest that *olrn-1(ky626) *has one or more functional AWC^OFF ^neurons and no AWC^ON ^neurons.

**Table 1:** *str-2 *expression in AWC in single and double mutants

	Percentage of animals (%)			
		
Strain	2AWC^ON^	1AWC^ON^/1AWC^OFF^	2AWC^OFF^	n
**(a) Wild type**	0	100	0	158
*olrn-1(ky626)*	0	1	99	198
*olrn-1(OE)*^a^	49	51	0	218
*nsy-4(OE)*^a^	69	29	2	92
*nsy-4(ky616)*	0	34	66	98
*nsy-5(OE)*^a^	57	42	1	111
*nsy-5(ky634)*	0	3	97	124
*unc-36(e251)*	84	16	0	73
*unc-2(lj1)*	44	44	12	165
*unc-2(e55)*	40	37	23	75
*unc-43(n1186)*	81	17	2	123
*tir-1(tm1111)*	69	31	0	149
*nsy-1(ky542)*	96	4	0	103
*sek-1(km4)*	74	23	3	125
*nsy-4(OE); olrn-1(ky626)*^a^	0	16	84	51^b^
*nsy-4(ky616); olrn-1(OE)*^a^	80	20	0	150^b^
*nsy-5(OE); olrn-1(ky626)*^a^	13	38	49	160^b^
*nsy-5(ky634); olrn-1(OE)*^a^	70	24	6	120^b^
*unc-36(e251);olrn-1(ky626)*	4	18	78	88^b^
*unc-2(lj1); olrn-1(ky626)*	0	0	100	110
*unc-2(e55); olrn-1(ky626)*	0	1	99	227
*unc-43(n1186); olrn-1(ky626)*	87	11	2	208
*tir-1(tm1111); olrn-1(ky626)*	20	80	0	115^b^
*nsy-1(ky542); olrn-1(ky626)*	95	3	2	217
*sek-1(km4); olrn-1(ky626)*	81	16	3	97
**(b) Wild type**	0	100	0	
*unc-2(lj1)*	63	33	4	72
*unc-36(e251)*	95	5	0	110
*egl-19(n582rf)*	0	100	0	107
*egl-19(ad695gf)*	0	100	0	125
*unc-2(lj1) egl-19(n582)*	97	3	0	204^b^
*unc-2(lj1) egl-19(ad695gf)*	0	5	95	128^b^
*unc-36(e251) egl-19(n582rf)*	88	11	3	64
*unc-36(e251) egl-19(ad695gf)*	21	54	25	73^b^

^a^Overexpression from extrachromosomal transgenic arrays *kyEx914 [odr-3::olrn-1b]*, *kyEx822 [odr-3::nsy-4]*, or *kyEx996 [nsy-5 genomic fragment]*. ^b^Significantly different from either single mutant (Chi square test, significance set at *P *< 0.025 to *P *< 0.006 as Bonferroni correction for multiple comparisons). Parts (a) and (b) were scored at different magnifications, resulting in slightly different values for *unc-2 *and *unc-36 *mutants.

A marker for the AWC^OFF ^neuron provided further evidence that *olrn-1 *disrupts AWC^OFF^/AWC^ON ^asymmetry. *srsx-3::GFP *is expressed bilaterally in the AWB neurons [14] and asymmetrically in one of the two AWC neurons (Table 2). In wild-type animals expressing an *srsx-3::GFP *transgene and a *str-2::dsRed *transgene, the neuron expressing *srsx-3::GFP *was invariably contralateral to the neuron expressing *str-2::dsRed *(Figure 1e), indicating that *srsx-3::GFP *is expressed in AWC^OFF^. This interpretation was confirmed by mutant analysis: *nsy-1(lf) *(ASK1/MAPKKK) and *unc-43(lf) *(CaMKII) failed to express *srsx-3::GFP *in either AWC neuron, consistent with their 2AWC^ON ^phenotype, and *unc-43(gf) *expressed *srsx-3::GFP *in both AWC neurons, consistent with its 2AWC^OFF ^phenotype (Table 2). *olrn-1(ky626) *mutants expressed *srsx-3::GFP *in both AWC neurons, suggesting that the neurons are both specified as AWC^OFF ^(Figure 1f).

**Table 2:** *srsx-3::GFP *is expressed in AWC^OFF^

	Percentage of animals (%) expressing *srsx-3 *in				
		
Genotype	2 AWC	1 AWC	0 AWC	2 AWB	n
*Ex(srsx-3::GFP)*	0	94	6	96	135
*nsy-1(ag3lf); Ex(srsx-3::GFP)*	0	0	100	93	89
*nsy-1(ok593lf); Ex(srsx-3::GFP)*	0	0	100	96	114
*unc-43(n408lf); Ex(srsx-3::GFP)*	0	0	100	97	144
*unc-43(n498gf); Ex(srsx-3::GFP)*	98	2	1	95	129

**Figure 2:**
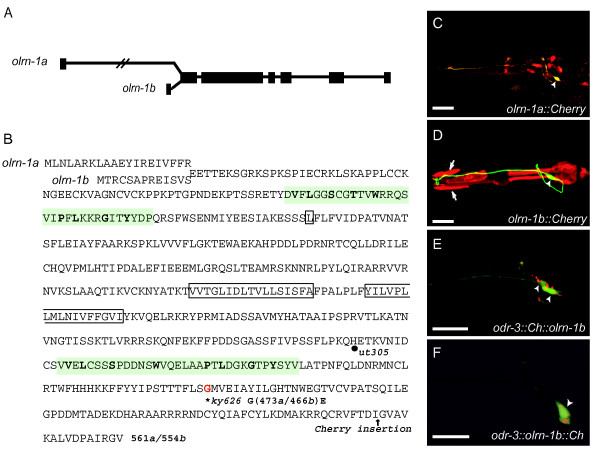
*olrn-1 *encodes a protein with Raw repeats and potential transmembrane domains. **(a) **Genomic structure of *olrn-1*, showing alternative first exons for *a *and *b *isoforms, whose 5' ends are separated by 3.8 kb. **(b) **Translation of *olrn-1*, showing alternative first exons for *olrn-1a *and *olrn-1b *isoforms. Two short repeats shared with *Drosophila *Raw are highlighted in green with conserved residues in bold type; potential transmembrane domains are boxed. The location of the splice acceptor site (fourth exon) mutated in *ut305 *and the residue mutated in *olrn-1(ky626) *(G 473a/466b E) are marked. Arrow marks the carboxy-terminal insertion site of Cherry in *odr-3::olrn-1b::Ch *(f). **(c) **Expression of *olrn-1a::Cherry *promoter fusion. Arrowhead indicates Cherry expression in AWC neuron expressing *str-2::GFP*. **(d) **Expression of *olrn-1b::Cherry *promoter fusion in non-neuronal cells. Arrows, hypodermal cells. The pharynx is a prominent site of expression. Arrowhead, no Cherry expression in AWC neuron expressing *str-2::GFP*. **(e) **Expression of amino-terminally tagged *odr-3::Cherry::olrn-1b *in an L4 *olrn-1(ky626) *animal. *odr-3::Cherry::olrn-1b *is excluded from the nucleus and is punctate in the axon and dendrite. **(f) **Expression of carboxy-terminally tagged *odr-3::olrn-1b::Cherry *in an L4 *ky626 *animal. *odr-3::olrn-1b::Cherry *is excluded from the nucleus, and is punctate in the axon and dendrite. Arrowheads, AWC cell bodies. Scale bars, 20 μm. Images are stacked confocal images.

*ky626 *was mapped using single nucleotide polymorphisms to a small region on X and was determined to be an allele of *olrn-1 *by failure to complement *olrn-1(ut305) *(see Materials and methods). *olrn-1(ut305) *corresponds to the C02C6.2 gene [7]. C02C6.2 has two isoforms, C02C6.2a and C02C6.2b (*olrn-1a *and *olrn-1b*, respectively), which differ in their first 13 or 20 amino acids due to the use of alternative first exons (Figure 2a,b) [15]. A G to A mutation was identified in *ky626 *mutants at position 473 in the *olrn-1a *isoform (position 466 in the *olrn-1b *isoform), resulting in a missense mutation (G → E) in both isoforms (Figure 2b). *olrn-1(ut305) *is mutated at the splice acceptor site of the fourth intron [7] and, like *olrn-1(ky626)*, results in a strong 2AWC^OFF ^phenotype (99% penetrant, n = 91). A strain with a deletion of C02C6.2 has a lethal phenotype (data not shown); this lethality may be the null phenotype of *olrn-1*, or it may result from a linked mutation in another gene.

Expression of *olrn-1 *cDNAs under the AWC-selective *odr-3 *promoter rescued the 2AWC^OFF ^phenotypes of *olrn-1(ky626) *mutants [7] (Table 3). Additionally, overexpression of *olrn-1b *in a wild-type background caused a 2AWC^ON ^phenotype (*olrn-1a *was not tested; Tables 1a and 3). These results indicate that high *olrn-1 *activity promotes the AWC^ON ^phenotype, support AWC as the likely site of *olrn-1 *function, and suggest that *ky626 *and *ut305 *are reduction-of-function alleles.

**Table 3:** Rescue of *olrn-1*, overexpression of *olrn-1*, and effect of mutations

			Percentage of animals (%)			
				
Test strain	Test clone(concentration)	Line	2AWC^ON^	1AWC^ON^/1AWC^OFF^	2AWC^OFF^	n
olrn-1	None		0	1	99	1,189
*olrn-1*	*odr-3::olrn-1b*	1	18	81	1	582
	(2.5 ng/μl)	2	1	77	22	218
*olrn-1*	*odr-3::olrn-1b*	1	48	51	1	639
	(5 ng/μl)	2	55	43	2	806
*olrn-1*	*odr-3::olrn-1b*	1	47	44	9	245
	(15 ng/μl)	2	52	41	8	293
WT	*odr-3::olrn-1b*	1	63	37	0	1528
	(15 ng/μl)	2	70	29	1	1282
WT	*odr-3::olrn-1b*	1	68	32	0	660
	(25 ng/μl)	2	70	30	0	781
*olrn-1*	*odr-3::olrn-1b::Ch*	1	81	16	3	73
	(15 ng/μl)	2	83	14	3	70
		3	76	22	2	88
*olrn-1*	*odr-3::olrn-1b(ΔrawR1)::Ch*	1	0	4	96	94
	(15 ng/μl)	2	0	1	99	91
		3	0	8	92	39
*olrn-1*	*odr-3::olrn-1b(ΔTM1,2)::Ch*	1	49	51	0	94
	(15 ng/μl)	2	53	46	1	96
*olrn-1*	*odr-3::olrn-1b(ΔrawR2)::Ch*	1	61	36	2	88
	(15 ng/μl)	2	81	19	0	83
		3	62	27	11	45
*olrn-1*	*odr-3::olrn-1b(G466E)::Ch*	1	6	86	7	81
	(15 ng/μl)	2	1	96	3	91
		3	5	91	5	43
*olrn-1*	*odr-3::olrn-1b(ΔRRRR)::Ch*	1	4	95	1	100
	(15 ng/μl)	2	20	78	2	110
		3	2	74	23	47
		4	7	88	5	152
*olrn-1*	*odr-3::olrn-1(ΔCterm)::Ch*	1	0	2	98	51
	(15 ng/μl)	2	0	0	100	88
		3	0	0	100	47

To establish the potential *olrn-1 *expression pattern, two regions upstream of *olrn-1 *were fused to coding sequences for the fluorescent protein *mCherry *[16]. The 3.8 kb region upstream of the *olrn-1a *start site was expressed in AWC neurons as well as ASG and BAG sensory neurons (Figure 2c). The 3.6 kb region upstream of the *olrn-1b *start site was expressed in the marginal cells of the pharynx, anterior hypodermal cells and the rectal gland cells (Figure 2d and data not shown). Although individual promoter fragments may not reproduce the entire *olrn-1 *expression pattern, these results suggest that *olrn-1 *may normally be expressed in AWC and in other cells.

### The first Raw repeat and a carboxy-terminal region are important for OLRN-1 function

OLRN-1 is a previously uncharacterized protein that is conserved along its entire length with related proteins from *Caenorhabditis remanei *and *Caenorhabditis briggsae*. It bears more distant similarity with the *Drosophila melanogaster *gene *raw *(or *cyrano*). *raw *restricts JNK signaling during dorsal closure of the fly embryo, and *raw *mutants have an embryonic dorsal-open phenotype resulting from abnormal cell migration, as well as nervous system defects [17]. The similarity between ORLN-1 and Raw is highest in two repeated domains of unknown function [17] (Figure 2b). OLRN-1 has a bipartite, highly hydrophobic region of approximately 40 amino acids at residues 264–280 and 288–304 that is likely to mediate membrane attachment; this domain is not present in Raw. One possibility is that these two hydrophobic domains form a hairpin-like transmembrane domain, so that both the amino and carboxyl termini of OLRN-1 face the cytoplasm.

The predicted OLRN-1B protein was tagged at its amino or carboxyl terminus by inserting *mCherry *into the *odr-3::olrn-1 *vector. Both amino- and carboxy-terminally tagged OLRN-1 rescued *olrn-1(ky626) *mutants (Table 3 and data not shown). The tagged OLRN-1b proteins were localized to punctate structures in the axon, dendrite, and cell body, but largely excluded from nuclei (Figure 2e,f).

**Figure 3:**
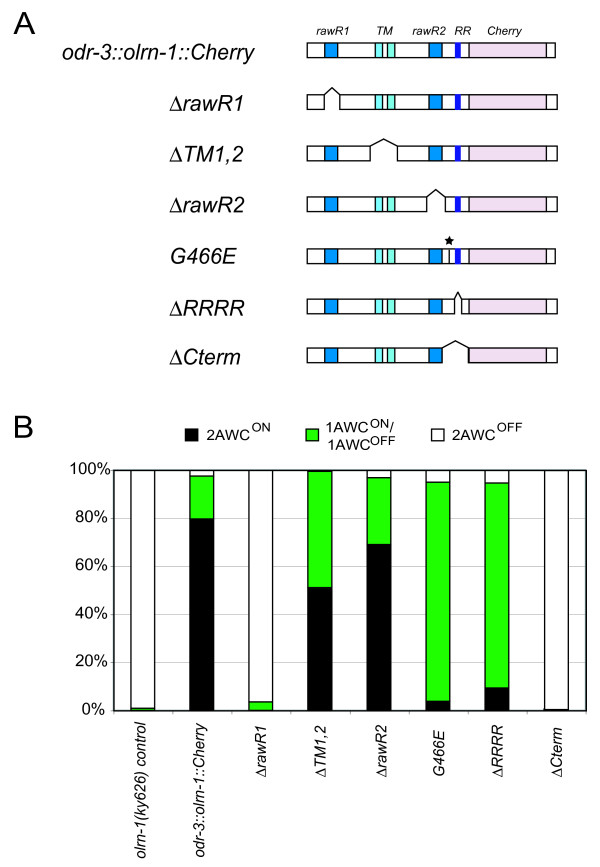
Structure-function analysis of *odr-3::olrn-1::Cherry*. **(a) **Mutants generated in *odr-3::olrn-1b::Cherry *affected Raw repeats (Δ*rawR1*, Δ*rawR2*), potential transmembrane domains (Δ*TM1,2*), the four adjacent arginines (Δ*RRRR*), and a carboxy-terminal region (Δ*Cterm*). The *ky626 *mutation (*G466E*) was also introduced. **(b) **Phenotypes of *ky626 *animals expressing *odr-3:olrn-1b::Cherry *transgenes. *olrn-1(ky626) *control is at left. All transgenes except Δ*Cterm *showed significant rescue compared to nontransgenic sibling controls; all transgenes except *rawR2 *were significantly less active than intact *odr-3::olrn-1b::Cherry *(*P *< 0.008 by Fisher exact test or Chi square test as appropriate; 0.008 was used as the significance level based on the conservative Bonferroni correction for six comparisons; n > 100 rescued animals per clone, from at least two independent transgenic lines that showed similar degrees of rescue (Table 3)).

Structure-function analysis of OLRN-1 was conducted to identify important domains of the protein (Figure 3a). Deletions in the *odr-3::olrn-1b::Cherry *rescuing clone were made to remove the predicted Raw repeats (Δ*rawR1*, Δ*rawR2*), the transmembrane domains (Δ*TM1,2*) and the region carboxy-terminal to the second Raw repeat (Δ*Cterm*). A set of four adjacent arginines reminiscent of a cleavage or nuclear localization signal was deleted from the carboxyl terminus (Δ*RRRR*). Finally, the G466E *ky626 *mutation was engineered into the full-length protein to examine the properties of the mutated protein. All of these Cherry-tagged mutant DNAs were cloned under the AWC-selective *odr-3 *promoter and introduced into *olrn-1(ky626) *mutants at 15 ng/μl, a concentration that resulted in a 2AWC^ON ^phenotype in approximately 80% of animals carrying a wild-type *odr-3::olrn-1b::mCherry *transgene (Figure 3b, Table 3). Deletion of the first Raw repeat (Δ*rawR1*) nearly eliminated the activity of *olrn-1*, as did deletion of the carboxy-terminal region (Δ*Cterm*). By contrast, deletions of the transmembrane domains (Δ*TM1,2*) or the second Raw repeat (Δ*rawR2*) did not greatly diminish the activity of *olrn-1 *(Figure 3b, Table 3). Transgenes carrying the deletion of the four arginines (Δ*RRRR*) and the G466E missense mutation were intermediate in activity. Δ*RRRR *and *G466E *transgenes were able to rescue *olrn-1(ky626)*, but did not cause the overexpression phenotype caused by the full-length *olrn-1 *transgene (Figure 3b). Their activity was similar to that of full-length *odr-3::olrn-1 *injected at six-fold lower DNA concentrations (Table 3). These results suggest that Δ*RRRR *and *G466E *mutations reduce the activity of the OLRN-1 protein.

The expression levels and localization of OLRN-1b were examined in *olrn-1(ky626) *animals bearing the mutated clones. All mutated clones produced comparable levels of OLRN-1::Cherry fluorescence in AWC, suggesting that the defective mutants made dysfunctional but stable proteins (Additional file 1). It was not possible to resolve the subcellular localization of OLRN-1 in embryos, but immediately after hatching the OLRN-1b::Cherry protein was present in the AWC cell body, axon and dendrite (Additional file 1a). All mutant proteins had similar localization to wild-type OLRN-1b::Cherry (Additional file 1b–g). Twelve hours later during the late L1/early L2 stage, wild-type OLRN-1b::Cherry and most mutant proteins were still localized to the AWC cell body, axon, and dendrite, but OLRN-1b(Δ*TM1,2*) was no longer detectable in axons (Additional file 1h–n).

### *olrn-1 *antagonizes calcium pathways in AWC signaling

The *olrn-1(ky626) *mutation and the *olrn-1(OE) *overexpressing transgene were combined with other mutations to ask how *olrn-1 *interacts with AWC asymmetry genes. We first examined the upstream signaling genes, the claudin-like *nsy-4 *and the innexin *nsy-5 *[9,10]. Loss-of-function mutations in *nsy-4*, *nsy-5 *and *olrn-1 *that caused 2AWC^OFF ^phenotypes were combined with overexpressing transgenes for *nsy-4*, *nsy-5 *and *olrn-1 *that caused 2AWC^ON ^phenotypes. In all combinations, the double mutants resembled the *olrn-1 *parent more closely than the *nsy-4 *or *nsy-5 *parent (Table 1a), but mixed phenotypes were observed. These results suggest that *olrn-1 *acts mainly at a step downstream of *nsy-4 *and *nsy-5*, but the absence of definitive null alleles of *nsy-4 *and *olrn-1 *limits this interpretation.

Loss-of-function mutations in the *unc-2 α *1 or *unc-36 α *2δ calcium channel subunits result in a strong (*unc-36*) or mixed (*unc-2*) 2AWC^ON ^phenotype. Both *unc-36 olrn-1 *double mutants and *unc-2 olrn-1 *double mutants resembled *olrn-1 *single mutants, with a high fraction of 2AWC^OFF ^animals (Table 1a). Different results were observed in double mutants between *olrn-1 *and loss-of-function mutations in the CaMKII/MAP kinase cascade – the kinase genes *unc-43 *(CaMKII), *nsy-1 *(MAPKKK), and *sek-1 *(MAPKK) [5,11,12]. Double mutants between *olrn-1 *and the three kinases invariably resembled the kinase mutants, with a strong 2AWC^ON ^phenotype (Table 1a). *tir-1 olrn-1 *double mutants had a mixed, nearly wild-type phenotype (Table 1a), but as neither gene has definitive null alleles, the significance of these results is unclear. These results suggest that *olrn-1 *acts between the calcium channels and the CaMKII/MAP kinase cassette (see Discussion).

### *unc-2 *CaV2 and *egl-19 *CaV1 calcium channel homologs act together in AWC asymmetry

The suggestion that *olrn-1 *acts at a genetic step near the voltage-activated calcium channel homolog *unc-2 *prompted a more detailed examination of *unc-2 *and *unc-36*. Previous studies showed that the putative CaV2 null mutant *unc-2(e55) *had a mixed AWC phenotype with 30–60% 2AWC^ON ^animals and 4–25% 2AWC^OFF ^animals (Table 1a) [5]. *unc-2(lj1)*, a second strong loss-of-function mutant, shared this mixed phenotype (Table 1b), but null mutants for the channel-associated α2δ subunit *unc-36 *had a strong 2AWC^ON ^phenotype [5] (Table 1b; see Materials and methods for molecular analysis of *unc-36(e251) *and *unc-2(e55)*). These results could be explained if multiple α1 subunits participate in AWC asymmetry, sharing the *unc-36 *α2δ subunit. The *C. elegans *genome encodes five proteins related to α1 subunits, including one CaV1 subunit and one CaV2 subunit. Null mutations in the CaV1 homolog *egl-19 *are embryonic lethal, and partial loss-of-function mutations have normal AWC asymmetry [18] (Table 1b). When *egl-19 *partial loss-of-function alleles were combined with null alleles of *unc-2*, the double mutants had a strong 2AWC^ON^phenotype reminiscent of *unc-36 *mutants (Table 1b). These results are consistent with partially redundant functions between *egl-19 *and *unc-2*, with both channels contributing to AWC asymmetry.

Calcium channels can serve as scaffolding proteins in addition to their ion-conducting properties. The ion-conducting properties of EGL-19 are affected by the gain-of-function mutation *egl-19(ad695gf)*, which decreases channel desensitization [18,19]. *egl-19(ad695gf) unc-2(lf) *mutants had a strong 2AWC^OFF^phenotype, the opposite phenotype from the *egl-19(lf) unc-2(lf) *mutants (Table 1b). This result suggests that the ion-conducting activity of EGL-19 contributes to its activity.

The strong 2AWC^OFF ^phenotype of *egl-19(ad695gf) unc-2(lf) *was not observed in *egl-19(ad695gf) *single mutants (Table 1b). Thus, *egl-19(gf) *has an activity in AWC that is masked by the normal activity of the *unc-2 *gene, but revealed when *unc-2 *is eliminated (see Discussion).

### *olrn-1 *acts in the future AWC^ON ^neuron, and *unc-2/unc-36 *act in the future AWC^OFF ^neuron, to coordinate AWC signaling

**Figure 4:**
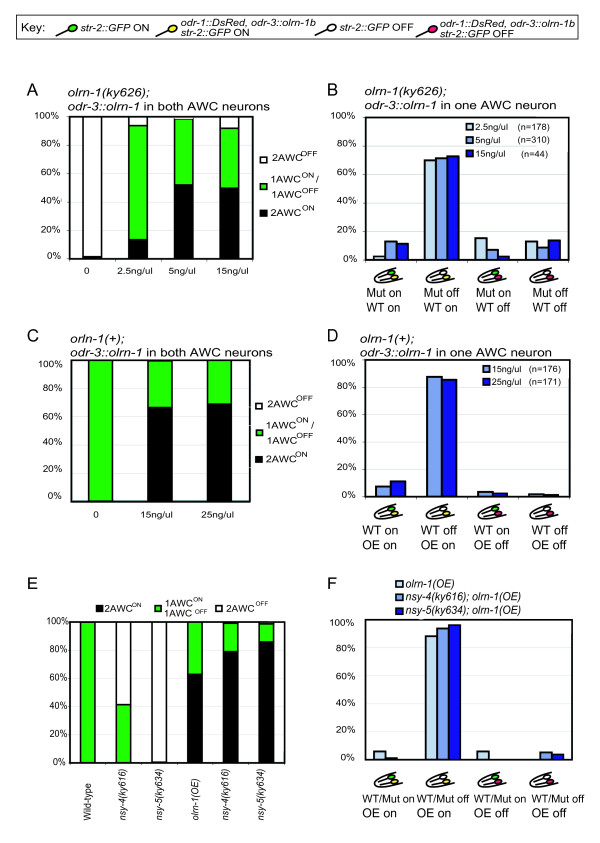
*olrn-1 *mosaic analysis. **(a) **Rescue of *olrn-1(ky626) *by *odr-3::olrn-1b *injected at 2.5 ng/μl, 5 ng/μl, or 15 ng/μl with *odr-1::DsRed*. **(b) **AWC phenotypes of mosaic *olrn-1(ky626) *animals that express *odr-3::olrn-1b odr-1::dsRed *transgene in one AWC. **(c) **Phenotypes in wild-type animals overexpressing *odr-3::olrn-1*b injected at 15 ng/μl or 25 ng/μl with *odr-1::DsRed*. **(d) **AWC phenotypes of wild-type mosaic animals that overexpress *odr-3::olrn-1b *in one AWC. For statistical analysis, see Materials and methods. **(e) **Phenotypes of *nsy-4 olrn-1(OE) *and *nsy-5 olrn-1(OE) *strains and controls. **(f) **AWC phenotypes of mosaic animals that express *odr-3::olrn-1b *in one AWC in *nsy-4 *or *nsy-5 *mutant backgrounds.

**Figure 5:**
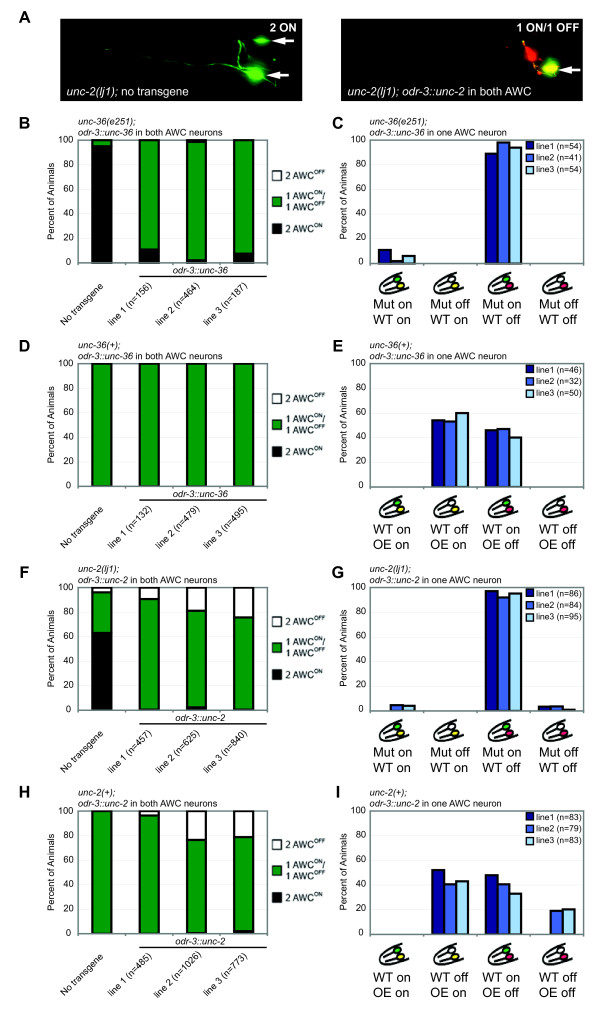
Mosaic analysis of the *unc-36/unc-2 *calcium channel genes. **(a) **Rescue of *unc-2(lj1) *by *[odr-3::unc-2, odr-1::dsRed] *array. Green, *str-2::GFP *expression; red, *odr-1::dsRED *expression. Arrows point to AWC^ON ^neurons. **(b) **Rescue of *unc-36(e251) *phenotypes in three *[odr-3::unc-36, odr-1::dsRed] *transgenic lines. **(c) **AWC phenotypes of *unc-36 *mosaic animals that express *odr-3::unc-36 *in one AWC. **(d) **The three *[odr-3::unc-36, odr-1::dsRed] *transgenes from (b) were introduced into a wild-type background. **(e) **AWC phenotypes of wild-type mosaic animals that overexpress *odr-3::unc-36 *in one AWC. **(f) **Rescue of *unc-2(lj1) *phenotypes in three *Ex [odr-3::unc-2, odr-1::dsRed] *transgenic lines. **(g) **AWC phenotypes of *unc-2 *mosaic animals that express *odr-3::unc-2 *in one AWC. **(h) **The three *[odr-3::unc-2, odr-1::dsRed] *transgenes from (f) were introduced into a wild-type background. **(i) **AWC phenotypes of wild-type mosaic animals that overexpress *odr-3::unc-2 *in one AWC. n, number of animals scored. For statistical analysis, see Materials and methods.

Genetic mosaic analysis is a useful approach for distinguishing between the two AWC neurons as they signal and respond to each other in development. Any gene in the asymmetry pathway could, in principle, function in the future AWC^OFF ^cell or in the future AWC^ON ^cell. Two kinds of results have been observed in previous genetic mosaic studies. The *nsy-4 *claudin and *nsy-5 *innexin genes act cell-autonomously to promote induction of AWC^ON^, and also act cell non-autonomously to prevent AWC^ON ^induction in the contralateral AWC [9,10]. At this signaling stage, each AWC appears to monitor the activity state of the other. By contrast, the kinases *unc-43 *and *nsy-1*, and the scaffold *tir-1 *act strictly cell-autonomously: a cell with high kinase activity becomes AWC^OFF^, and a cell with low kinase activity becomes AWC^ON^[11,12]. At this execution stage, the decision has been made and the AWCs are independent. To understand how the decision is made, we used genetic mosaic analysis to examine animals in which the two AWCs had different levels of *olrn-1*,*unc-2 *and *unc-36 *gene activity (Figures 4 and 5). Unstable extrachromosomal arrays containing the AWC marker *odr-1::DsRed *and either *odr-3::olrn-1*,* odr-3::unc-2*, or *odr-3::unc-36 *test plasmids were introduced into strains with stable expression of the AWC^ON ^marker *str-2::GFP *(Figures 4a,c and 5b,d,f,h). Random loss of the arrays from one AWC was detected using the *odr-1::DsRed *marker, and then both AWC neurons were scored for the expression of the *str-2::GFP *AWC^ON ^marker. The methods for these experiments were similar to those used in previous studies; control experiments indicate that the promoters and markers do not affect AWC asymmetry (see Materials and methods) [10-12].

In an *olrn-1 *mutant background, most mosaic animals with a single rescued AWC had the wild-type, asymmetric phenotype: the rescued cell became AWC^ON ^and the mutant cell became AWC^OFF ^(Figure 4b). This result suggests that *olrn-1 *acts cell autonomously in the future AWC^ON ^cell to induce its identity. In wild-type animals overexpressing *odr-3::olrn-1 *transgenes, most animals that lost the transgene in one AWC also had a wild-type asymmetric AWC phenotype. In these animals, the cell overexpressing *olrn-1 *nearly always became AWC^ON^, and the wild-type contralateral cell nearly always became AWC^OFF ^(Figure 4d). This behavior is unlike the behavior of fully wild-type animals in which each cell becomes AWC^ON ^or AWC^OFF ^at equal frequencies. The wild-type mosaics suggest that a cell with higher *olrn-1 *expression can prevent the contralateral cell from becoming AWC^ON^, and therefore implicate *olrn-1 *in feedback between AWCs. Very similar results were previously obtained in mosaic analysis of *nsy-4 *and *nsy-5 *[9,10].

The results described above might be confounded by *nsy-4- *and *nsy-5*-dependent signaling between AWCs. To separate cell-intrinsic functions of *olrn-1 *from possible network functions, we generated mosaics overexpressing OLRN-1 in one AWC in *nsy-4 *and *nsy-5 *mutants (Figure 4e,f). In these experiments,*olrn-1(OE) *behaved exactly as it did in the wild-type background, specifically converting the *olrn-1-*expressing neuron to AWC^ON ^(Figure 4f). These results suggest that *olrn-1 *functions independently of, and most likely downstream of,*nsy-4 *and *nsy-5 *in AWC^ON^.

Similar rescue and mosaic overexpression experiments were conducted with the calcium channel genes *unc-36 *(α2δ subunit) and *unc-2 *(CaV2 α1 subunit). Expression of either gene under the AWC-selective *odr-3 *promoter rescued AWC asymmetry of the corresponding mutant (see Materials and methods; Figure 5a,b,f). Thus, the calcium channels are likely to function within AWC neurons. A mild gain-of-function phenotype was observed with *odr-3::unc-2*, but not with *odr-3::unc-36 *(Figure 5d,h).

For both *unc-36 *and *unc-2*, mosaic animals with a single rescued AWC had the wild-type asymmetric phenotype: the rescued cell always became AWC^OFF^, and the mutant cell always became AWC^ON ^(Figure 5c,g). This result was substantially different from the result with downstream kinases such as *unc-43*, where a single rescued AWC randomly became AWC^ON ^or AWC^OFF^, and the mutant cell always became AWC^ON ^[12]. The difference suggests that *unc-36 *and *unc-2 *influence the coordinated AWC^ON^/AWC^OFF ^decision, whereas *unc-43 *and other kinases act to execute a decision that has already been made. The rescue of the *unc-2 *mutant cell in these mosaics is particularly informative. *unc-2 *has an incompletely penetrant phenotype; in *unc-2 *controls, 33% of animals have one AWC^OFF ^neuron, and 4% have two AWC^OFF ^neurons, so in total approximately 20% of all *unc-2 *mutant AWCs became AWC^OFF ^(Table 1b). In *unc-2 *mosaics with one rescued AWC, fewer than 5% of the *unc-2(-) *AWCs became AWC^OFF^, indicating that the *unc-2(-) *AWC neuron was affected by the rescued AWC on the contralateral side.

Only a mild overexpression phenotype was observed upon introduction of *odr-3::unc-2 *into wild-type animals (Figure 5h), suggesting that the tightly regulated calcium channels may be relatively resistant to variations in expression levels. In mosaic animals in which only one AWC overexpressed *odr-3::unc-36 *or *odr-3::unc-2 *in a wild-type background, the overexpressing AWCs or the contralateral AWCs were equally likely to become AWC^ON ^or AWC^OFF ^(Figure 5e,i). These results indicate that unlike *nsy-4*,*nsy-5*, and *olrn-1*, relative *unc-2 *and *unc-36 *expression levels are not critical to the AWC^ON^/AWC^OFF ^decision.

## Discussion

This analysis adds two genes to the pathway for AWC asymmetry: the gene *olrn-1*, and the *C. elegans *CaV1 homolog *egl-19*, whose cooperation with *unc-2 *explains the weak phenotype of the *unc-2 *(CaV2) mutant. These genes and other genes in the AWC asymmetry pathway have been classified in three ways: double mutant analysis, which can reveal biological regulatory relationships; targeted rescue and mosaic analysis to determine the essential cellular site of expression; and detailed mosaic analysis to determine whether expression of the gene in one AWC affects the contralateral AWC. Combining these three approaches suggests that the coordinated decision between AWC^ON ^and AWC^OFF ^occurs at the interface between the calcium channels (UNC-2/UNC-36/EGL-19) and OLRN-1.

In *C. elegans *AWC neurons, *olrn-1 *has genetically defined functions that are similar to those of the innexin gene *nsy-5 *and the claudin/calcium channel γ subunit gene *nsy-4*. All three genes are required for the induction of AWC^ON^, and all have similar cell-autonomous and non-autonomous effects on AWC in mosaic analysis [9,10].*olrn-1 *overexpression induced AWC^ON ^cell-autonomously in *nsy-4 *and *nsy-5 *mutants, suggesting that *olrn-1 *acts downstream of these two genes or independently of them in AWC^ON^. The nature of any *olrn-1 *regulation by the upstream genes is unknown. There were no obvious effects of *olrn-1 *mutations on tagged NSY-4 or NSY-5 proteins in AWC, nor were there obvious effects of *nsy-4 *or *nsy-5 *on tagged OLRN-1 protein (data not shown).

*olrn-1 *mutations were epistatic to null mutations in the calcium channel genes *unc-2 *and *unc-36*, whereas calcium channel null mutations are epistatic to *nsy-4 *and *nsy-5 *[9,10]. The behavior of *olrn-1 *in these double mutants supports the suggestion that it acts at a later step in signaling than *nsy-4 *and *nsy-5*. Epistasis analysis does not provide detailed molecular mechanisms, and some conclusions are not firm when null alleles are unavailable. However, since the *unc-2 *and *unc-36 *mutations are molecular nulls, the epistasis result proves that these channel genes are not essential for *olrn-1 *activity.

**Figure 6:**
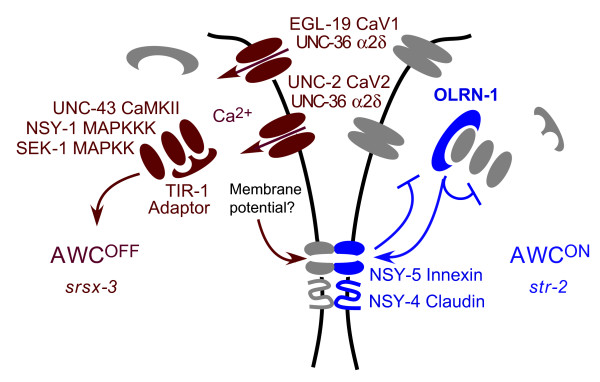
Model for calcium channel function and OLRN-1 in the AWC^ON^/AWC^OFF^decision. All genes are expressed both in the left and in the right AWCs; color is used to indicate the cell in which each gene product is more active. The future AWC^OFF ^transmits a signal to AWC^ON ^via NSY-5 gap junctions between AWC and other cells and NSY-4 claudins. This signal might be membrane potential. In AWC^ON^, the signal suppresses the UNC-2 (CaV2) and EGL-19 (CaV1) voltage-activated calcium channels and allows high OLRN-1 activity. OLRN-1 inhibits the UNC-43 (CaMKII)/NSY-1/SEK-1 kinase cascade cell-autonomously within AWC^ON^. A feedback signal from the calcium channels and OLRN-1 is transmitted from AWC^ON ^back to AWC^OFF^.

The kinase mutations *unc-43 (*CaMKII), *nsy-1 *(ASK1/MAPKKK), and *sek-1 *(MAPKK) were fully epistatic to *olrn-1 *mutations. Thus, at a genetic level, *olrn-1 *may prevent *unc-2 *from activating the CamKII homolog *unc-43 *in the future AWC^ON^. In molecular terms, this might mean that OLRN-1 inhibits calmodulin (which would act at this step) or that OLRN-1 prevents calcium from UNC-2 channels from activating UNC-43, perhaps by binding the channel or the kinase (Figure 6). There is no evidence for direct interactions between these proteins, and many other possibilities exist. *olrn-1 *is related to *Drosophila raw/cyrano*, which is required for epithelial movements that drive dorsal closure of the fly embryo, for epithelial morphogenesis, and for neuronal development [17,20]. An *olrn-1 *domain that is similar to *raw *is needed for full *olrn-1 *activity, supporting the significance of the homology. The direct targets of Raw are unknown, but *raw *mutants have excessive phospho-Jnk during dorsal closure, suggesting that Raw inhibits Jnk MAP kinase pathways [17]. This biochemical evidence that Raw is a kinase inhibitor parallels our genetic conclusion that *olrn-1 *directly or indirectly inhibits the kinase pathway consisting of *unc-43 *(CaMKII), *nsy-1 *(ASK1, a p38/Jnk MAPKKK), and *sek-1 *(MAPKK).

The analysis of *egl-19 *CaV1 mutations underscores the importance of calcium channels in AWC asymmetry. *unc-2 *CaV2 mutants have a weak and mixed phenotype, raising initial doubts about the significance of the channel, but the highly penetrant 2AWC^ON ^phenotype of *egl-19 unc-2 *double mutants suggests that calcium entry through voltage-activated calcium channels is essential for specification of AWC^OFF^. The strong, opposite 2AWC^OFF ^phenotype of the *egl-19(gf) unc-2(lf) *double mutant further suggests that sufficient calcium entry through EGL-19 can act instructively to specify AWC^OFF^. This phenotype was not observed in *egl-19(gf) *single mutants, suggesting that *unc-2 *inhibits the activity of *egl-19(gf)*. CaV channels generate calcium and voltage signals, and are subject to calcium- and voltage-dependent activation and inactivation, so there are many levels at which channel cross-regulation could take place [21].

The phenotype of *unc-36 *in AWC asymmetry suggests that this CaV α2δ subunit promotes the activity of both *unc-2 *and *egl-19 *α1 subunits. Previous calcium imaging studies of *C. elegans *pharyngeal muscles suggested that *unc-36 *inhibits *egl-19 *[19], whereas calcium imaging in mechanosensory neurons suggested that *unc-36 *activates *egl-19 *[22]. We suggest that both previous observations are correct, and that in pharyngeal muscles, as in AWC, *unc-2/unc-36 *channels inhibit *egl-19 *or *egl-19/unc-36 *channels.

A model for the functions of *olrn-1*,* unc-2*, and *unc-36 *in the signaling pathway, based on this work and prior work, is presented in Figure 6. Induction of AWC^ON ^from an AWC^OFF^-like ground state requires cooperation between the innexin gene *nsy-5*, which assembles a multicellular gap junction network and preferentially induces AWCR to the AWC^ON ^state, and the claudin/γ-subunit like *nsy-4*, which preferentially induces AWCL to the AWC^ON ^state [9,10]. Tight junctions (which contain claudins) and gap junctions (which are composed of innexins) potentiate one anothers' activity in epithelia, providing a possible analogy for the *nsy-4/nsy-5 *cooperation in AWC [23]. A signal must be transmitted by this multicellular network; the strong involvement of the voltage-regulated calcium channel homologs *unc-2*,*egl-19*, and *unc-36 *in AWC asymmetry suggests that the signal regulates membrane potential. Voltage changes are efficiently transmitted through gap junctions, whereas calcium is poorly diffusible and is, therefore, transmitted inefficiently. Thus, voltage signals from UNC-2, EGL-19, and possibly other channels could be transmitted from AWC^OFF ^to AWC^ON ^via gap junctions. At least two other voltage-regulated channels, the potassium channels SLO-1 and EGL-2, also affect AWC asymmetry [5,24]. The appeal of this model is that voltage-regulated channels such as *unc-2/unc-36 *could act both to generate a signal in one AWC and to detect the signal in the contralateral AWC.

The coordinated decision to form one AWC^ON ^and one AWC^OFF ^requires a symmetry-breaking event. Like many genes in the AWC asymmetry pathway, *unc-2/unc-36 *activity is predicted to be high in one AWC, and low in the other; unlike other genes, there are plausible mechanisms by which a symmetry-breaking event could differentially regulate calcium channels. An interesting example is provided by the pacemaker cells of the vertebrate heart, which are found in the sinoatrial (SA) node. Gap junctions and voltage-activated calcium channels are essential to the synchronization of SA pacemaker cells and the generation of a coherent heartbeat [25]. Isolated SA cells have rhythmic action potentials that are driven by calcium channels and other conductances. When two SA cells come into contact, they form gap junctions that lead to synchronization of the two cells, at a rhythm that is dominated by the faster, or leader, cell. Two synchronized SA cells would appear to be similar, but in fact, the result of their synchronization is a coupling of membrane potential and an uncoupling of individual conductances within the two cells [26]. During the diastolic period between heartbeats, the leader cell has an ongoing inward current, while the follower cell has an outward current [26]. In other words, the leader cell experiences inward currents both at the beginning of the calcium action potential and in the long period between action potentials; the follower cell experiences inward currents only during the action potential. As calcium-activated signaling pathways are exquisitely sensitive to the temporal pattern of calcium signals [27,28], different patterns of inward calcium currents in two cells have the potential to create sustained differences between them.

We suggest that in isolation, both AWCs have spontaneous activity sufficient to maintain CaMKII activity and the AWC^OFF ^state. When gap junctions form via NSY-5, the spontaneous activity of the AWCs is coupled, and by analogy to the SA node, one cell leads and the other follows. The leader cell maintains ongoing calcium entry and becomes AWC^OFF^; gap junction coupling reduces calcium entry into the follower cell, and it becomes AWC^ON^. In this model, the calcium channels have both an effector function (calcium entry and activation of CaMKII) and a signaling function (altering membrane potential). We speculate that similar mechanisms may operate in many developing nervous systems during the transient period that gap junctions are prominent. Since it acts at a similar step, *olrn-1 *could affect either the propagation of the signal or its effectiveness in the responding cell.

Gap junctions, claudins, and membrane potential affect left-right asymmetry of the *Xenopus *body axis, suggesting a possible molecular similarity between vertebrate asymmetry and the pathways that regulate *C. elegans *AWC neurons [29-31]. Although the later nodal/Shh pathways used in vertebrate left-right patterning are different from those used in AWCs, there may be hidden similarities that remain to be discovered. The left-right asymmetry of the human brain is more variable than the human body plan; left-handedness and reversed lateralization of language areas are much more common than inversion of the internal organs [32]. Lateralized neurological disorders such as migraine headaches and Rasmussen encephalitis randomly affect one side of the brain, providing indirect evidence of variably asymmetric brain functions [33,34]. An asymmetric migraine syndrome in humans is caused by mutations in CaV2 calcium channels, which are orthologs of *unc-2 *[33]. Further analysis of asymmetric brain function in humans may reveal unexpected connections with the asymmetric nervous system of *C. elegans*.

## Materials and methods

### Strains

Wild-type strains were *C. elegans *variety Bristol, strain N2. The CB4856 strain was used for mapping *olrn-1 *[35]. Strains were maintained by standard methods [36].

Germline transformation was carried out as previously described [37]. Co-injection markers were *ofm-1::GFP*,*ofm-1::RFP *or *elt-2::GFP*. Integrated transgenes used in this study included *kyIs140 I [str-2::GFP, lin-15(+)]*, *kyIs323 II [str-2::GFP, ofm-1::GFP]*, *zdIs5 [mec-4::GFP]*. Mutations used in this study included *LG (linkage group) I: nsy-5(ky634)*, *LGII: nsy-1(ky542)*, *nsy-1(ag3)*,*nsy-1(ok593)*,* LGIII: tir-1(tm1111)*, *unc-36(e251)*, *LGIV: egl-19 (n582)*,* egl-19(ad695gf)*,* nsy-4(ky616)*,* unc-43(n1186)*,* unc-43(n408)*,* unc-43(n498gf)*, *LGX: olrn-1(ky626)*,*olrn-1(ut305)*, *unc-2 (lj1)*,*unc-2(e55)*, *sek-1(km4)*.

Transgenes maintained as extrachromosomal arrays included the following lines used for mosaic analysis: kyEx914 (line 1) & kyEx918 (line 2) [odr-3::olrn-1b 15 ng/ul, odr-1::dsRed 7.5 ng/ul, ofm-1::gfp 20 ng/ul], kyEx1072 (line 1) & kyEx1074 (line 2) [odr-3::olrn-1b 5 ng/ul, odr-1::dsRed 7.5 ng/ul, ofm-1::gfp 20 ng/ul], kyEx1097 (line 1) & kyEx1098 (line 2) [odr-3::olrn-1b 2.5 ng/ul, odr-1::dsRed 7.5 ng/ul, elt-2::GFP 10 ng/ul], kyEx1102 (line 1) & kyEx1103 (line 2) [odr-3::olrn-1b 25 ng/ul, odr-1::dsRed 7.5 ng/ul, elt-2::GFP 5 ng/ul]. For unc-2 and unc-36 mosaics, the same extrachromosomal arrays were examined in wild-type and mutant backgrounds: kyEx1628 (line 1) kyEx1629 (line 2) and kyEx1630 (line 3) [odr-3::unc-2 20 ng/ul, odr-1::RFP 2.5 ng/ul, ofm-1::GFP 10 ng/ul]; kyEx1229 (line 1) kyEx1387 (line 2) and kyEx1388 (line 3) [odr-3::unc-36 20 ng/ul, odr-1::RFP 2.5 ng/ul, elt-2::GFP 10 ng/ul].

Additional transgenic arrays were kyEx822 [odr-3::nsy-4a 75 ng/ul, ofm-1::GFP 20 ng/ul], kyEx996 [18.5 kb nsy-5 PCR fragment 13 ng/ul, odr-1::DsRed 12.5 ng/ul, ofm-1::GFP 25 ng/ul], kyEx1075 [srsx-3::GFP 10 ng/ul, str-2::DsRed 50 ng/ul, elt-2::gfp 10 ng/ul], kyEx1096 [odr-1::dsRed 7.5 ng/ul, elt-2::GFP 10 ng/ul], kyEx1182 [odr-3::olrn-1b::Ch 5 ng/ul, ofm-1::GFP 15 ng/ul], kyEx1320 [olrn-1a::Ch 25 ng/ul, ofm-1::GFP 15 ng/ul], kyEx1315 [olrn-1b::Ch 50 ng/ul, elt-2::GFP 10 ng/ul], kyEx1310 [odr-3::Ch::olrn-1b 25 ng/ul, elt-2::GFP 10 ng/ul].

The following arrays were used in the deletion analysis: *odr-3::olrn-1b::Ch *= *kyEx1318 *(line 1), *kyEx1319 *(line 2), *kyEx1317 *(line 3); *odr-3::olrn-1bΔ rawR1::Ch *= *kyEx1337 *(line 1), *kyEx1345 *(line 2), *kyEx1344 *(line 3); *odr-3::olrn-1bΔ TM1,2::Ch *= *kyEx1297 *(line 1), *kyEx1298 *(line 2); *odr-3::olrn-1bΔ rawR2::Ch *= *kyEx1338 *(line 1), *kyEx1339 *(line 2), *kyEx1340 *(line 3); *odr-3::olrn-1b(G466E)::Ch = kyEx1358 *(line 1), *kyEx1357 *(line 2), *kyEx1359 *(line 3); *odr-3::olrn-1b(ΔRRRR)::Ch = kyEx1332 *(line 1), *kyEx1296 *(line 2), *kyEx1300 *(line 3), *kyEx1299 *(line 4); *odr-3::olrn-1bΔ Cterm::Ch = kyEx1382 *(line 1), *kyEx1352 *(line 2), *kyEx1351 *(line 3). These transgenes were injected at 15 ng/μl with 10–15 ng/μl of *ofm-1::GFP *co-injection marker.

### Isolation and characterization of *olrn-1(ky626)*

*kyIs140 I *animals were subjected to ethyl methane sulfonate (EMS) mutagenesis according to standard procedures [38]. A chemotaxis enrichment was used to isolate F2 animals that sensed 2,3 pentanedione (an AWC^OFF^-sensed odorant) but failed to sense butanone (an AWC^ON^-sensed odorant) [10]. F2 mutants that failed to migrate to butanone were screened for the AWC^OFF ^*str-2::GFP *phenotype using a fluorescence dissecting microscope. The failure to express *str-2::GFP *was confirmed using 400× magnification under a compound microscope. Additional rounds of screening were done without the behavioral enrichment.

*olrn-1(ky626) *was mapped on LGX between single nucleotide polymorphisms pkP6166 (physical position X: 14678988) and pkP6172 (physical position X:17707311) using the CB4856 strain [35]. A complementation test between *ky626 *and *ut305 *resulted in a failure to complement: 93.5% of *ky626/ut305 *(n = 138) animals at 25°C were 2AWC^OFF^. *olrn-1(ut305) *has previously been shown to correspond to C02C6.2 (X:15539330–15546729) [7]. To identify the *olrn-1(ky626) *mutation, genomic coding regions of C02C6.2 were amplified by PCR and sequenced on both strands. The *olrn-1(ky626) *mutation was a G → A transition, resulting in a G → E missense mutation at residue 473 in the *olrn-1a *isoform, and position 466 in the *olrn-1b *isoform.

### Molecular biology

#### Identification of unc-2(e55) and unc-36(e251) mutations

Resequencing *unc-2(e55) *revealed that the stop mutation originally assigned to residue 458 was actually present at reside 511 of the T02C5.5b gene model (Q>stop nonsense mutation), but supported the identification of *e55 *as a strong loss-of-function mutation.

Sequencing of *unc-36(e251) *revealed that the *unc-36(e251) *mutation was a G → A transition, resulting in a Trp>stop nonsense mutation at residue 496 in the C50C3.9a gene model. This mutation should truncate the UNC-36 protein immediately after the vWA domain.

#### odr-3::unc-2

*unc-2 *cDNAs were obtained by PCR from a *C. elegans *cDNA library using primers flanking the open reading frame T02C5.5b. Due to toxicity of the full-length cDNAs in bacteria, they were maintained as minigenes with a synthetic intron interrupting their coding regions. When expressed from the pan-neuronal *H20 *promoter, the *unc-2 *minigene rescued the uncoordinated phenotype of *unc-2(lj1)*. The minigene was subcloned behind the *odr-3 *promoter for mosaic analysis.

#### odr-3::unc-36

*unc-36 *cDNAs were obtained by PCR from a *C. elegans *cDNA library using primers flanking the open reading frame C50C3.9a. Expression of the cDNA under 2 kb of *unc-36 *upstream region rescued the uncoordinated phenotype of *unc-36(e251)*. The cDNA was subcloned behind the *odr-3 *promoter for mosaic analysis.

#### odr-3::olrn-1b

*odr-3::olrn-1b *was constructed by inserting a *Kpn*I-C02C6.2b-*Sma*I fragment into the pPD49.26 vector at the *Kpn*I and *Eco*RV sites. A *Kpn*I-C02C6.2b-*Apa*I fragment from this clone was then inserted downstream of the *odr-3 *promoter in the *odr-3::GFP *vector [39] removing the green fluorescent protein (GFP).

#### odr-3::mCherry::orln-1b & odr-3::olrn-1b::mCherry

The mCherry coding sequence was amplified using primers with linkers on each side of mCherry, and subcloned into either the 5' *Nhe*I site upstream of the *olrn-1b *start site or an internal *Eco*RV site in the carboxyl terminus in the *odr-3::olrn-1b *vector. This insertion site was carboxy-terminal to the second Raw repeat domain (*rawR2*).

#### odr-3::olrn-1b deletions

To make the domain deletions described in Figure 3, the *odr-3::olrn-1b::Cherry *vector was deleted at residues 77–108 (Δ*rawR1*), 265–304 (Δ*TM1,2*), 396–428 (Δ*rawR2*), 510–513 (Δ*RRRR*), and 429–539 (Δ*C-term*) by PCR overlap extension with suitable primers [40,41]. The same technique was used to introduce the G466E mutation.

#### olrn-1a::mCherry and olrn-1b::mCherry

3.8 kb 5' to the *olrn-1a *start site and 3.6 kb 5' to the *olrn-1b *start site were subcloned into the pSM-mCherry vector. The second clone represented the entire intron between the alternative first exons of the OLRN-1 isoforms.

### Genetic mosaic analysis

Loss of function mosaic analysis was performed on six independent lines with unstable extrachromosomal transgenic arrays *[odr-3:olrn-1b, odr-1::dsRed] *in a *kyIs140 I, olrn-1(ky626) X *mutant. Gain-of-function mosaic analysis was performed on four independent lines in a *kyIs140 I *wild-type strain. Each data point in Figure 4 represents combined data from two independent lines injected with the same concentration of DNA. The presence or absence of the transgene in mosaics was inferred by the presence of *odr-1::dsRed*, which is expressed in AWC and AWB neurons. The *str-2::GFP *expression phenotype in mosaic cells was examined under a compound scope at 100–400×. Previous experiments have used a similar strategy [9-12]. Statistical analysis was performed for mutant mosaics rescued at 2.5 or 5 ng/ul of *odr-3:olrn-1b*, and for wild-type mosaics injected with 15 or 25 ng/μl of *odr-3:olrn-1b*, to test: the null hypothesis that both AWC neurons behaved as independent units, purely as predicted by the proportions in the non-mosaic controls; the null hypothesis that the DsRed-positive (rescued) AWC behaved purely as predicted by the rescued controls; and the null hypothesis that the DsRed-negative (non-rescued) AWC behaved purely as predicted by the non-rescued controls. In all cases, results were different from the null hypothesis at *P *< 0.001 by Chi square test or Fisher exact test as appropriate, using the calculator at [42]. Both the rescued AWC and the non-rescued AWC in *olrn-1; odr-3::olrn-1b *mosaics became AWC^ON ^more often than predicted by the null hypothesis. Thus, there is both autonomous and non-autonomous rescue of *olrn-1*. In wild-type mosaics overexpressing *olrn-1*, the overexpressing AWC became AWC^ON ^more often than predicted by the null hypothesis, and the wild-type AWC became AWC^OFF ^more often than predicted.

In rare *olrn-1 *mosaic animals, the transgene was lost in both AWC neurons but retained in either or both AWB neurons. These animals did not express *str-2::GFP *(n = 5 animals), suggesting that *olrn-1 *expression in AWC accounts for its major role in AWC asymmetry.

Mosaic analysis of *olrn-1 *in *nsy-4 *or *nsy-5 *mutants was performed on a single line per genetic background. A single unstable extrachromosomal array was examined in wild-type, *nsy-4(ky616) *or *nsy-5(ky634) *backgrounds bearing stable integrated *str-2::GFP *transgenes.

Loss-of-function mosaic analysis for *unc-2 *was performed on three independent lines with unstable extrachromosomal transgenic arrays *[odr-3::unc-2, odr-1::dsRed] *in a *kyIs140 [str-2::GFP] I; unc-2(lj1) X *mutant. Gain-of-function mosaic analysis for *unc-2 *was performed on three independent lines in the *kyIs140 I *strain. Loss-of-function mosaic analysis for *unc-36 *was performed on three independent lines with unstable extrachromosomal transgenic arrays *[odr-3::unc-36-SL2-CFP, odr-1::dsRed] *in a *kyIs140 I; unc-36(e251) III *mutant. Gain-of-function mosaic analysis for *unc-36 *was performed on three independent lines in the *kyIs140 *strain. Loss of the transgene was inferred by loss of the co-injection marker *odr-1::dsRed *in AWC neurons. Results from all lines were combined for statistical analysis, which was performed as described above for *olrn-1*. In all cases the null hypothesis that the two AWCs behaved as independent units could be excluded at *P *< 0.001. Both the rescued AWC and the non-rescued AWC in *unc-2; odr-3::unc-2 *mosaics were strongly affected by the contralateral cell – the rescued AWC became AWC^OFF ^more often than predicted by the null hypothesis, and the non-rescued AWC became AWC^ON ^more often than predicted (*P *< 0.001 in both cases). Thus, a single *unc-2(+) *AWC can bias both AWC neurons. In wild-type mosaics overexpressing *unc-2*, the wild-type AWC became AWC^OFF ^more often than predicted by the null hypothesis (*P *= 0.003), but the overexpressing AWC was not affected by the wild-type AWC (*P *= 0.1943). In *unc-36; odr-3::unc-36 *mosaics, the wild-type AWC became AWC^OFF ^more often than predicted by the null hypothesis (*P *< 0.001), but the mutant AWC was not significantly affected (*P *= 0.075). There was no significant effect of *unc-36 *overexpression in a wild-type background.

### Microscopy

To visualize early L1s, 50–100 gravid adults were picked and allowed to lay eggs overnight. Adults and hatched larvae were washed off the plate, and eggs were allowed to hatch for three hours. L1 larvae were examined on a compound microscope at 400–630× magnification or used for confocal microscopy. To obtain late L1/early L2 animals, approximately 50–60 gravid adults were picked to a plate and allowed to lay eggs for 3 hours, after which the adults were removed from the plate. Progeny were examined 30 hours later under the confocal microscope.

## Competing interests

The author(s) declare that they have no competing interests.

## Authors' contributions

SLBH, YS, and CIB designed the experiments, and SLBH and YS performed the experiments. MKV isolated the *olrn-1(ky626) *mutant, and IT, TI, and IK cloned *olrn-1*. AvdL and PS characterized the *srsx-3 *reporter. SLBH and CIB wrote the paper.

## Supplementary Material

Additional File 1 Subcellular localization of wild-type and mutant *olrn-1b::Cherry *proteins. Confocal images of *odr-3::olrn-1b::Cherry *proteins in *olrn-1(ky626) *animals at (**a-g**) 0–3 hours after hatching and (**h-n**) L1/L2 stage, 20 hours after hatching. Notched arrowheads indicate AWC axons, flat arrowheads indicate AWC cell bodies. The diagrams show the approximate size and disposition of AWC neurons in the images (anterior is at left). **(a-g) **Two AWCs are visible in most images; **(h-n) **only one AWC is visible in most images, but both AWCs have similar OLRN-1b:Cherry levels. Scale bars are 10 μm. Click here for file
