# Supplementary figures and images for: Left-right olfactory asymmetry results from antagonistic functions of voltage-activated calcium channels and the Raw repeat protein OLRN-1 in C. elegans
Source: Neural Dev. 2007 Nov 6;2:24. doi: 10.1186/1749-8104-2-24 (PMC2213652; doi:10.1186/1749-8104-2-24)

Supplementary Figure 1

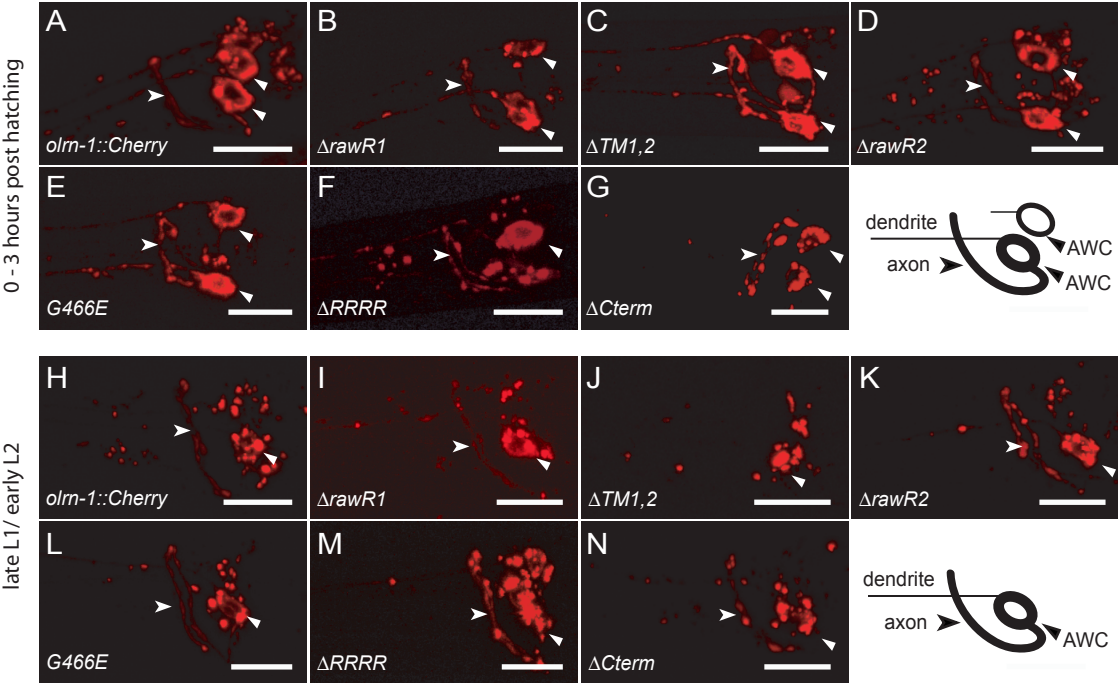

Supplement: Additional File 1 — Subcellular localization of wild-type and mutant olrn-1b::Cherry proteins. Confocal images of odr-3::olrn-1b::Cherry proteins in olrn-1(ky626) animals at (a-g) 0–3 hours after hatching and (h-n) L1/L2 stage, 20 hours after hatching. Notched arrowheads indicate AWC axons, flat arrowheads indicate AWC cell bodies. The diagrams show the approximate size and disposition of AWC neurons in the images (anterior is at left). (a-g) Two AWCs are visible in most images; (h-n) only one AWC is visible in most images, but both AWCs have similar OLRN-1b:Cherry levels. Scale bars are 10 μm. [file 1749-8104-2-24-S1.pdf]
